# P-2313. Multinational, Retrospective Study Assessing Outcomes and Healthcare Resource Utilization in Hematopoietic Stem Cell Transplant Recipients with Cytomegalovirus Infection and Refractory, Resistance, or Intolerance to Anti-Cytomegalovirus Treatments: Analysis of Impact of Viremia Clearance on Outcomes

**DOI:** 10.1093/ofid/ofae631.2465

**Published:** 2025-01-29

**Authors:** Kimberly Davis, Ines Mendonça, Tien Bo

**Affiliations:** Takeda Development Center Americas, Inc., Lexington, Massachusetts; CTI Clinical Trial & Consulting Services, Lisbon, Lisboa, Portugal; Takeda Development Center Americas, Inc., Lexington, Massachusetts

## Abstract

**Background:**

Cytomegalovirus (CMV) infection is a risk factor for morbidity and mortality in hematopoietic stem cell transplant (HSCT) recipients. This real-world analysis describes the impact of viremia clearance on outcomes in HSCT recipients with refractory, resistant or intolerant (RRI) CMV infection to anti-CMV agents.

**Figure:**
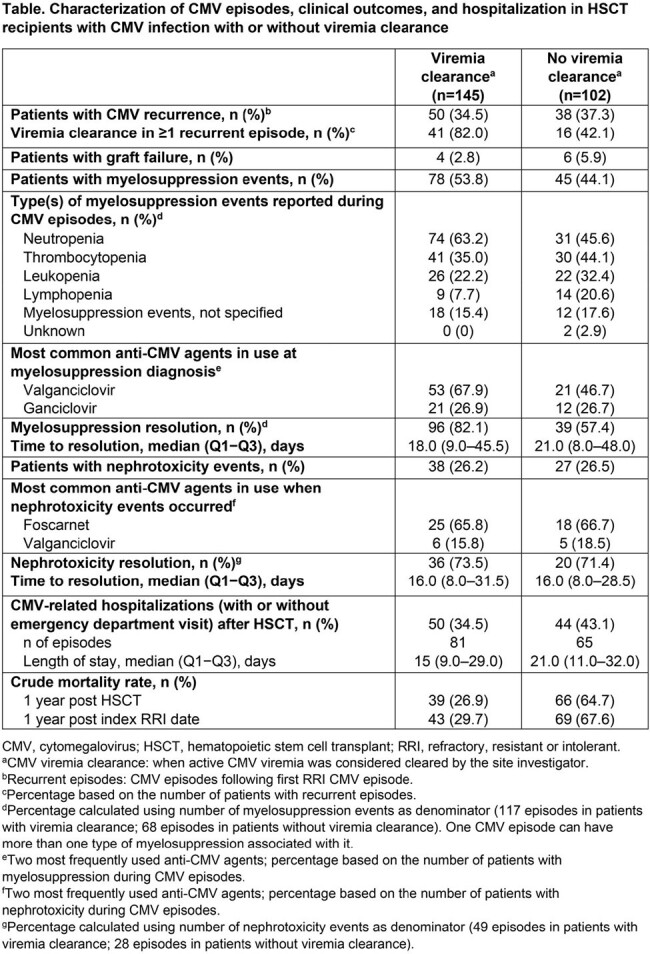

**Methods:**

This multicenter, retrospective chart review study included adult HSCT recipients with RRI CMV infection. Data between 2014 and 2021 were collected from 14 centers in Europe and the USA. The analysis descriptively compared demographics, patient characteristics, clinical outcomes, and hospitalizations in patients who did versus did not achieve viremia clearance during the first RRI CMV episode.

**Results:**

Among 247 patients with a first RRI CMV episode, 145 (59%) achieved viremia clearance and 102 (41%) did not. In patients with vs without viremia clearance, respectively, median age was 53 vs 60 years, and 46% vs 58% were male. Of the 145 and 102 first RRI CMV episodes with vs without viremia clearance, respectively, 52% vs 75% were identified as refractory, 61% vs 31% as intolerant, and 3% vs 9% as resistant (categories not mutually exclusive); 9% vs 15% of patients developed tissue invasive disease. CMV recurrence occurred in 35% vs 37% and graft failure in 3% vs 6% of patients with vs without viremia clearance, respectively (Table). Myelosuppression and nephrotoxicity events during CMV episodes were reported in 54% vs 44% and 26% vs 27% of patients with vs without viremia clearance, respectively (Table). CMV-related hospitalizations with/without emergency department visits occurred in 35% of patients with viremia clearance vs 43% of patients without viremia clearance (Table). Notably, the overall mortality within 1 year of RRI identification in patients without viremia clearance was higher than in those with viremia clearance (68% vs 30%, respectively).

**Conclusion:**

These results highlight the high disease burden in HSCT recipients with RRI CMV infection. In this difficult-to-treat population, the mortality rate was higher in patients without viremia clearance than in patients with viremia clearance.

**Disclosures:**

Kimberly Davis, RPh, MS, Takeda: Employee|Takeda: Stocks/Bonds (Public Company) Ines Mendonça, MSBiostats, CTI Clinical Trial & Consulting Services: Employee (paid to provide consulting services including medical writing and statistical analysis) Tien Bo, PharmD, Takeda: Employee|Takeda: Stocks/Bonds (Public Company)

